# Outcomes of elective head and neck confirmed or suspected cancer surgery during the COVID-19 pandemic

**DOI:** 10.1007/s00405-020-06194-2

**Published:** 2020-07-15

**Authors:** Sabrina Brar, Enyi Ofo, Nicholas Hyde, Dae Kim, Tunde Odutoye, David Allin, Aleix Rovira

**Affiliations:** 1 ENT Department, St George’s University Hospital, London, UK; 2 Oral and Maxillofacial Surgery Department, St George’s University Hospital, London, UK

**Keywords:** Head and neck surgery, COVID-19, Coronavirus, Mortality

## Abstract

**Purpose:**

To analyse the complication outcomes of COVID-19 negative patients undergoing elective head and neck surgery during the COVID-19 pandemic.

**Methods:**

This was a retrospective case review of all patients undergoing elective head and neck surgery for confirmed or suspected head and neck cancer.

**Results:**

There were no mortalities recorded in the cohort of patients analysed. At 30 days, pulmonary complications had occurred in 4 patients (9%). None of these were related to COVID infection.

**Conclusion:**

With careful pre-operative screening of patients for COVID-19 and post-operative care in a COVID-19 clean ward, head and neck surgery can proceed safely during the epidemic. This data could help to minimise delay in treatment by allowing a greater number of elective head and neck cancer operations to proceed.

## Introduction

Coronavirus (COVID-19), the novel disease caused by the severe acute respiratory syndrome coronavirus 2 (SARS-CoV-2), was declared a pandemic by the World Health Organisation in March 2020 [1]. There have been over 7,250,000 confirmed cases worldwide and to date, over 400,000 people have died as a result of the disease [1]. The disease outbreak has tested the resilience of global healthcare services.

Our institution is a tertiary centre for head and neck cancer and documented its first coronavirus deaths on the 12th March 2020. Within 8 days, the total number of patient deaths in this hospital accounted for 1/5th of all deaths recorded in London [2]. By mid-April, London was the worst hit region in the United Kingdom, recording the highest age-adjusted mortality rate in the country (85.7 per 100,000 deaths related to COVID-19) [3].

In response to the pandemic, a dramatic and unprecedented change to clinical practice has necessarily been implemented. Primarily, this has been to increase inpatient capacity to meet the demands created by the acute surge in COVID-19 patients, many of whom require ventilated beds in an intensive care setting. This requires staff re-allocation away from normal surgical duties. Furthermore, early published data have suggested that the 30-day mortality and morbidity were significantly higher in patients undergoing both elective and emergency surgery when SARS-CoV-2 infection was confirmed perioperatively [4]. For these reasons, NHS England and other professional bodies worldwide have issued guidance on how best to prioritise patients undergoing surgery during this time. As a result of the above, our institution acted to limit all ‘non-essential’ surgery, thereby minimising the risk of in-hospital COVID-19 transmission, as well as any post-operative pulmonary complications that may ensue [5–7].

Sadly, delaying elective surgery will have a significant negative impact on both patients and healthcare systems worldwide. Whilst many elective operations can be delayed, the treatment of many head and neck cancers cannot be safely deferred. Any delay in such patients’ cancer must be weighed against associated increased morbidity and mortality.

Herein, we present the complication outcomes of patients undergoing elective head and neck surgery for confirmed or suspected cancer during the COVID-19 pandemic in a hospital treating a high volume of COVID-19 patients. This study reports on the 30-day mortality and morbidity of elective head and neck surgery, including both the rate of pulmonary and non-COVID-19-related complications. This data provides a baseline from which hospitals can plan their post-pandemic surgical recovery. There has been only one similar small study published in the literature that investigated the outcome of three patients undergoing elective head and neck surgery during the COVID-19 pandemic [8].

## Methods

### Study design

This was a retrospective case review of all patients undergoing elective head and neck surgery for confirmed or suspected head and neck cancer.

### Study population

All patients undergoing elective head and neck surgery for confirmed or suspected cancer at our institution over an 8-week period (23rd March–20th May 2020) were included in this study. The hospital’s Standard Operative Procedure (SOP) (dated 1st May 2020) stated that all patients undergoing elective surgery had to confirm if they were unwell, exhibited any COVID-19 symptoms or had been in close contact with a person with symptoms. Patients were advised to self-isolate for 14 days prior to surgery. A nasopharyngeal swab was taken 48 h prior to admission. On the day of surgery, patients underwent chest radiography (CXR), which was reported by a radiologist. Perioperatively, patients remained in a designated COVID ‘green’ part of the hospital, where all patients had a COVID-negative swab and were asymptomatic for COVID symptoms. Prior to 1st May 2020, there was no formal SOP but our unit’s practice was similar.

### Exclusion criteria

Patients undergoing procedures under local anaesthetic, trauma/emergency surgery or other otolaryngological procedures were not included.

### Variables

The primary outcome measure was 30-day mortality of patients undergoing surgery. The secondary outcome measure was 30-day morbidity including pulmonary and other post-operative complications. Pulmonary complications were defined as atelectasis, pneumonia, acute respiratory distress syndrome and post-operative ventilation (non-invasive/invasive) that was not expected as part of the normal post-operative course. Post-operative complications were graded using the head and neck modified Clavien–Dindo classification [8]. Only routine anonymised data were collected.

## Results

A total of 47 patients were included in the final analysis. Patient demographics including age, sex, co-morbidities and ASA (American Society of Anaesthesiologists physical status classification) were recorded [9] (Table 1). Pre-surgery COVID symptoms and status were analysed—no patients reported any pre-operative COVID symptoms (defined as cough and/or fever, shortness of breath and myalgia). Accordingly, all of the patients were assumed to be COVID-19 negative; this was supported by a negative swab in 81% of the patients (Table 2). The remaining 19% of patients were operated on prior to our hospital’s SOP, which stated that a swab and CXR must be taken prior to surgery.

**Table 1:** Baseline demographic characteristics

	Patient numbers	30-Day mortality (no. of patients)	30-Day pulmonary complications (no. of patients)	30-Day morbidity (no. of patients)
Total patients	47	0	4 (9%)	8 (17%)
Sex				
Male	31 (66%)	0	3 (6%)	4 (9%)
Female	16 (34%)	0	1 (2%)	4 (9%)
Age (years)				
< 29	2 (4%)	0	0	0
30–49	13 (28%)	0	0	3 (6%)
50–69	24 (51%)	0	3 (6%)	4 (9%)
> 70	8 (17%)	0	1 (2%)	1 (2%)
Number of co-morbidities				
0	13 (28%)	0	0	3 (6%)
1	15 (32%)	0	2 (4%)	2 (4%)
2 or more	19 (40%)	0	2(4%)	3 (6%)
ASA grade				
1	13 (28%)	0	0	1 (2%)
2	26 (55%)	0	2 (4%)	6 (13%)
3	6 (13%)	0	1 (2%)	1 (2%)
4	2 (4%)	0	1 (2%)	0
Symptoms at admission				
None	47 (100%)	0	0	0
Symptoms	0	0	0	0
Cancer stage				
Diagnostic surgery	19 (40%)	0	1 (2%)	4 (9%)
1	11 (23%)	0	1 (2%)	0
2	4 (9%)	0	1 (2%)	1 (2%)
3	2 (4%)	0	0	0
4	11 (23%)	0	1 (2%)	3 (6%)
Operation				
Panendoscopy ± tonsillectomy ± dental extractions	17 (36%)	0	1 (2%)	4 (8%)
Wide local excision tongue/buccal mucosa ± neck dissection	11 (23%)	0	2 (4%)	1 (2%)
Nasal biopsy ± debulking	7 (15%)	0	1 (2%)	
Total thyroidectomy + selective neck dissection	4 (9%)	0		1 (2%)
Parotidectomy ± neck dissection	3 (6%)	0		
Trans oral robotic surgery + selective neck dissection	1 (2%)	0		1 (2%)
Selective neck dissection	1 (2%)	0		
Excision lymph node	1 (2%)	0		
Major head and neck resection with free flap reconstruction	1 (2%)	0		1 (2%)
Right partial maxillectomy + obturator insertion	1 (2%)	0

**Table 2:** Negative COVID-19 status confirmed with nasal swab and/or CXR

COVID-19 status	Total patients
Negative (swab and CXR)	22 (47%)
Negative (swab or CXR)	38 (81%)
No swab or CXR	9 (19%)

There were no mortalities recorded in the cohort of patients analysed (Table 1). At 30 days, pulmonary complications had occurred in four patients (9%). None of these were related to COVID infection (Table 3). Three of the patients who suffered pulmonary complications were male; they were all over the age of 50 with at least one co-morbidity that included being a current/ex-smoker.

**Table 3:** 30-day pulmonary complications following elective head and neck surgery

Sex	Age	Co-morbidities	ASA	COVID-19 negative swab	Normal CXR	Operation	Pulmonary complication
M	54	Asthma, HTN, ex-smoker, COPD	3	Nil	Nil	Wide local excision tongue, selective neck dissection, radial forearm free flap	Bacterial pneumonia
M	68	Ex-smoker	2	Nil	Yes	Wide local excision buccal mucosa, selective neck dissection	Atelectasis
F	72	Smoker, OA, HTN, COPD	4	Negative (2)	Yes	Endoscopic debulking of left sinonasal mucosal melanoma	Bacterial pneumonia
M	62	Smoker	2	Negative (1)	Yes	Panendoscopy	Saturations dropped 88–92 (non-diagnosed COPD)

30-day morbidity for non-pulmonary complications was recorded in eight patients (17%) (Table 4). They were equally distributed between males and females. 88% of the patients were aged 30–69.

**Table 4:** 30-day post-operative complications following elective head and neck surgery

Sex	Age	Co-morbidities	ASA	COVID-19 Negative swab	Normal CXR	Post-operative complication (using the head and neck modified Clavien–Dindo classification [8]
M	67	T2DM, ex-smoker	3	Negative (2)	Normal	I
M	68	Ex-smoker	2	Nil	Normal	II
M	43	Nil	1	Negative (1)	Nil	II
F	46	Nil	2	Nil	Nil	II
M	70	AF	2	Negative (1)	Nil	III
F	37	Nil	2	Negative (1)	Normal	I
F	67	Smoker, GORD, high cholesterol	2	Nil	Normal	II
M	54	Ex-smoker, HTN	2	Negative (1)	Normal	II

## Discussion

The COVID-19 pandemic has drastically changed clinical practice worldwide and the delivery of routine elective surgery has been disrupted to protect patients and healthcare workers. It has been estimated that 28,404,603 operations globally could be postponed due to the COVID-19 pandemic [10]. Currently, there is no vaccination or cure for COVID-19. Results from one recent English study revealed that only 6.78% of the general population were positive for COVID-19 antibodies and so it is likely that COVID-19 will continue to be problematic for the foreseeable future [11]. Key considerations must be taken to allow for the resumption of elective services to help address the significant backlog of surgical work and potential harm to patients waiting for their delayed treatment [12].

Initial analysis of patients undergoing elective surgery that were asymptomatic and not known to be infected with SARS-CoV-2 in China during the COVID-19 pandemic suggested that surgery may exacerbate COVID-19 disease progression. Lei et al. [13] demonstrated a mortality rate of 20.5% in patients undergoing various planned operations and all patients went on to develop pneumonia. 30-day mortality and morbidity have been found to be significantly higher (23.7% mortality and 50.9% pulmonary complications) in patients undergoing surgery (elective and emergency), when SARS-CoV-2 infection is confirmed perioperatively [4]. The most vulnerable patients have been identified as males over the age of 70 with co-morbidities, those undergoing cancer or other major surgery and patients requiring emergency operations [4]. Patients with cancer are at an increased risk of contracting COVID-19 compared with non-cancer patients [14] and the rate of fatality amongst this patient cohort has been found to be 5.6% compared with 2.3% for the average population [15].

The impact of operating on non-coronavirus elective head and neck patients has not been notably analysed. However, as a result of published studies such as Lei et al. [13] and a paucity of local data regarding patient safety outcomes, thresholds for performing surgery during these times have been rightly scrutinised. This led to the NHS England guideline for the management of patients requiring surgery during the COVID-19 crisis. This document describes five priority levels and their corresponding action, to be decided by a consultant lead within each speciality [16]. At the peak of the COVID-19 pandemic, only priority level 1a, 1b and 2 patients were permitted to undergo surgery nationally. This was supported by BAHNO, ENT UK and BAETS [17]. A comparable approach has been adopted by institutions in other countries such as France, USA and Australia [18–20] and has also been recommended by a consortium of international clinical trials units, member organisations of the Head and Neck Cancer International Group [21].

The multidisciplinary management of head and neck cancer patients is complex. This cohort of patients is usually older and co-morbid, often with tobacco-related respiratory diseases, and therefore has a higher risk of developing severe post-operative complications both with and without SARS-CoV-2 exposure and infection [8, 9]. Delaying treatment is, however, associated with disease progression and increased patient anxiety, which can negatively impact patient survival outcomes [9].

Our study analysed patient data over an 8-week period during the peak of the COVID-19 pandemic in a hospital treating a high number of patients infected with SARS-CoV-2. The study identified that, in our case series, there was no 30-day mortalities related to COVID-19 or other causes in patients asymptomatic for SARS-CoV-2 infection undergoing elective head and neck surgery. 81% of the patients were confirmed to be negative for SARS-CoV-2 infection perioperatively through swab testing or CXR. The remaining 19% did not have confirmed evidence of COVID-19 status in their documentation; however, they underwent surgery as no symptoms of COVID-19 or contact with anyone with symptoms was reported pre-operatively.

Furthermore, the study did not demonstrate any notable risk of 30-day pulmonary complications—9% of the patients analysed had a documented lung complication post-operatively. No complication was COVID-19 related; no patients tested positive for SARS-CoV-2 infection post-operatively. At the time of analysis, no patient remained in hospital following surgery. This has a significant implication for clinical practice, as it is evidence from a London teaching hospital and acute medical admission and trauma unit at the epicentre of the COVID-19 pandemic. The data can be interpreted and used to balance the risk of delaying elective surgery versus proceeding with operating on head and neck cancer patients.

### Study limitations

There are limitations to recognise when interpreting the data presented in this study. Although all patients undergoing elective head and neck surgery were included, the sample size was relatively limited. Baseline patient demographics were analysed; however, ethnicity was not included. This is important because black, Asian and minority ethnic groups have been found to have a markedly higher vulnerability and risk of mortality from COVID-19 [22].

Lastly, a nasopharyngeal swab, and/or CXR was used as means of determining pre-operative SARS-CoV-2 infection status. The benefit of testing patients however relies on the accuracy of the test and current literature states that the exact sensitivity and specificity for diagnosing infection with SARS-CoV-2 in unknown [23]. The recent introduction of antibody testing may form an important part of pre-surgery assessment, in conjunction with or replacing the nasopharyngeal COVID-19 swab. Mounting an antibody response is however host-dependent and studies have shown that the average time a patient takes to seroconvert SARS-CoV-2 infection is 7–11 days [24]. Antibody testing is, therefore, not useful to determine acute infection status. Similarly, it is not yet known whether people who have been infected with and recovered from SARS-CoV-2 are fully immune from further infection; however, in combination with a nasopharyngeal swab, antibody testing could form part of the COVID-19 ‘pandemic suppression campaign’ [24], enabling hospitals to move forward and perform surgery in the absence of a COVID-19 vaccination or cure [24].

## Conclusion

This study looks at surgical 30-day mortality and morbidity rates in COVID-19 negative patients undergoing elective diagnostic and major therapeutic head and neck oncological surgery. There was no recorded mortality or post-operative COVID-19 related complications in our case series. Our results suggest that, with careful pre-operative screening of patients for COVID-19 and post-operative care in a COVID-19 clean ward, head and neck surgery can proceed safely during the epidemic. This data could help to minimise the delay in treatment by allowing a greater number of elective head and neck cancer operations to proceed.

## Data Availability

The data that support the findings of this study are available from the corresponding author upon reasonable request.
